# Case report: a missed case of chronic Q fever infective endocarditis demonstrating the ongoing diagnostic challenges

**DOI:** 10.1099/acmi.0.000628.v3

**Published:** 2023-11-24

**Authors:** Victoria Grey, Peter Simos, Naomi Runnegar, Jared Eisemann

**Affiliations:** ^1^​ Infection Management Services, Princess Alexandra Hospital, Brisbane, Australia; ^2^​ School of Medicine, University of Queensland, Brisbane, Australia; ^3^​ Infection Management Services, Gold Coast University Hospital, Gold Coast, Australia; ^4^​ Infection Management Services, Mater Hospital, Brisbane, Australia

**Keywords:** dual pathology, infective endocarditis, Q fever

## Abstract

Diagnosis of chronic Q fever is often difficult for clinicians, particularly in the presence of a second pathology. In addition to the chronic constitutional symptoms, the most common manifestations of chronic Q fever include infective endocarditis and endovascular infection. We describe a case of prosthetic valve infective endocarditis caused by both *Streptococcus sanguinis* and *Coxiella burnetti* on a background of a previous aortic graft and bioprosthetic aortic valve replacement 2 years earlier. The diagnosis of chronic Q fever infective endocarditis was delayed because the significance of the abnormal valve histology from the patient’s previous surgery was initially overlooked. It was only after the patient had relapsed on appropriate therapy for the *S. sanguinis* prosthetic valve endocarditis that a subsequent review of the operative valve histology, along with the patient’s epidemiological risk factors, led to consideration of an additional culture-negative cause for infective endocarditis. Histological examination of the valve tissue had shown exophytic fibrin vegetations and acute inflammation. Further clinical assessment revealed previous exposure to Q fever and *C. burnetti* DNA was detected via polymerase chain reaction on the valve tissue. Q fever infective endocarditis must be considered if valves are inflamed or have vegetations with a subsequent negative culture. It should also still be considered in the presence of an alternative bacteraemia if the patient has risk factors for exposure.

## Data Summary

No data were generated during this research or are required for the work to be reproduced.

## Background

*Coxiella burnetti,* a small intracellular Gram-negative bacterium, is the aetiological agent of Q fever, a global zoonotic disease with a broad clinical spectrum ranging from either asymptomatic or mild disease to life-threatening infection [1, 2]. Animals, in particular small ruminants, act as reservoirs for the bacterium, with transmission occurring through close contact with contaminated tissue, in particular the birth products of these animals [1, 3]. Ticks are also important vectors of transmission [4]. Airborne transmission is prominent and the organism is highly infectious; even distant contact with infected animals can lead to infection [5]. Australia has an age-standardized Q fever seroprevalence of 5.6 % and it is most commonly found in Queensland and New South Wales [3, 6].

Acute Q fever can present with flu-like symptoms, pneumonia or hepatitis, or be asymptomatic [3]. Approximately 1–5 % of acutely infected humans will go on to develop chronic Q fever, which can manifest years after primary infection [7]. Early chronic Q fever can present with constitutional symptoms, including fevers, night sweats and weight loss, but more severe sequelae, including infective endocarditis and vascular infection, are associated with chronic Q fever, with arthritis and osteomyelitis also described in the literature [3, 7].

Serology is the gold standard for diagnosing chronic Q fever, with indirect immunofluorescence preferred [8]. Phase 2 antibodies are the first to appear, followed by phase 1 antibodies as a result of antigen phase variation [9]. This helps determine the stage of infection; when phase 1 IgG antibodies titres are ≥800 or are higher than phase 2 IgG levels it is highly predictive of chronic Q fever [10]. The time to the development of chronic infection varies from months to years after acute infection [11].

The diagnosis of chronic Q fever can be challenging due to its protean manifestations and indirect diagnosis using serology [12]. Further, despite endocarditis being a common clinical manifestation, the infection itself is still rare, making it easy to overlook [13]. A previous case series has described patients presenting with endocarditis caused by *C. burnetti* and also another concomitant bacterial pathogen [14, 15]. There have also been case reports of patients with acute bacterial endocarditis occurring concurrently with acute Q fever [15]. We present a case report of a patient who was initially diagnosed with *Streptococcus sanguinis* prosthetic valve infective endocarditis and then subsequently, on retrospective assessment, concomitant Q fever infective endocarditis that had been present for over 2 years.

## Case report

In December 2021, a 68 year old male presented with a 7 month history of worsening fatigue, weight loss (approximately 20 kg), and night sweats. His past medical history included a previous bioprosthetic aortic valve replacement, aortic root replacement and coronary artery bypass graft in 2019 for moderate bicuspid aortic valve stenosis, aortic root dilatation and left anterior descending coronary artery disease. Further to this, he had a history of atrial fibrillation, likely amiodarone-induced thyrotoxicosis, hypertension, dyslipidaemia and gout. He had no risk factors for infective endocarditis, including no recent dental procedures or intravenous drug use. He was born in Australia and had never lived overseas. There was no history of regular animal contact, but 3 years previously he had visited his brother’s cattle farm in Wylie Creek in New South Wales, Australia. Incidentally, his brother had previously been diagnosed with Q fever. Physical examination showed no peripheral stigmata of infective endocarditis, such as splinter haemorrhages, Osler nodes or Janeway lesions, no appreciable cardiac murmurs, no spinal tenderness and no palpable hepatosplenomegaly. Two sets of blood cultures grew *S. sanguinis* [with a penicillin G minimum inhibitory concentration (MIC) of 0.125] and he had resolution of his bacteraemia within 24 h of commencement of intravenous antibiotics. Transoesophageal echocardiogram showed mild mitral regurgitation but otherwise no significant abnormality that would require surgery and surprisingly no evidence of valve vegetations or root replacement dysfunction. An abdominal CT scan showed isolated splenomegaly without any other focus of infection. A dental review was unremarkable. A colonoscopy was performed, which did not reveal a gastrointestinal source for his bacteraemia. A diagnosis of presumed *S. sanguinis* endovascular infection was made. Initial treatment with intravenous benzylpenicillin was complicated by a suspected penicillin-induced acute interstitial nephritis and the patient was changed to intravenous ceftriaxone. He was discharged on home intravenous 2 g daily ceftriaxone via our local outpatient antimicrobial service. This was initially planned for 6 weeks with a PET scheduled to further evaluate for the presence of prosthetic infection. However, he relapsed with fevers after 3 weeks with the subsequent PET scan showing FDG uptake around his aortic root concerning for infection.

Due to the previous history of his brother having Q fever and the fact that the patient had visited his farm, Q fever serology was requested. The Q fever phase 2 EIA was reactive with a phase 1 IgG titre of 1 : 40 925 and a phase 2 IgG titre of 1 : 10 240 using immunofluorescence at our local laboratory. The positive serology in conjunction with his CT/PET findings met the criteria for definite Q fever vascular infection as per the Dutch consensus guidelines [7]. Q fever polymerase chain reaction (PCR) was also detected on serum.

The pathological and histological findings from the patient’s initial cardiac surgery in 2019 were reviewed, with further consideration given to the possibility of pre-existing Q fever infection prior to his surgery. The histology had shown fibrin exophytic vegetations on the aortic valve, but when the culture had remained negative after 5 days, their significance was overlooked and further culture-negative diagnostics were not requested. Following this repeat review 2 years later, *C. burnetti* DNA was detected by PCR on the aortic tissue from the original operation. This confirmed the initially missed diagnosis of chronic Q fever endocarditis.

The patient was commenced on doxycycline and hydroxychloroquine for a planned minimum duration of 24 months, as well as completing his 6 weeks of directed antibiotic therapy for the *S. sanguinis *. At the time of writing, he has remained well and has required no further cardiac intervention.

## Discussion

We describe a patient with initial presumed *S. sanguinis* aortic graft infection given his cardiac history and absence of an alternative source, who then relapsed with persistent culture-negative fevers. This led to a co-existing diagnosis of chronic Q fever infective endocarditis based on CT/PET findings and serological evidence of chronic Q fever. In light of these findings, retrospective review of valve tissue from the patient’s initial cardiac surgery 2 years previously detected Q fever by PCR. This case demonstrates that Q fever infective endocarditis should still be considered as a dual diagnosis in the presence of a separate bacteraemia if risk factors for an exposure exist. This case also highlights that Q fever infective endocarditis can be a difficult diagnosis to make and delays in diagnosis and treatment can result in further complications. Had Q fever endocarditis been considered earlier in this patient, he may have avoided cardiothoracic surgery.

A previous retrospective review of 439 patients with chronic Q fever in the Netherlands found that the mortality rate was as high as 38 % for cases of proven Q fever [16]. Mortality was higher in patients who had developed complications; the highest fatality rates were seen amongst patients with both a vascular and an endocarditis focus (33 %) [16]. Q fever infective endocarditis has high rates of relapse after antimicrobial cessation and requires a longer duration of treatment when a prosthetic valve is involved [17]. It usually involves the mitral and the aortic valves [17]. Infection involving a prosthetic valve is associated with higher risk of relapse and requires a longer duration of treatment [17]. Given these potential consequences of infection, it is crucial for clinicians to be aware of this infection as well as diagnostic methods.

There are a variety of diagnostic techniques that can be used to help facilitate an earlier diagnosis. The main diagnostic technique relies on serology, which can also differentiate between acute and chronic Q fever [7]. Seroconversion usually occurs within 1 month, but can take up to 6 months [18]. Given seroconversion can take up to 4 weeks after infection, negative serology early on in the disease course does not rule out infection. The indirect immunofluorescence assay (IFA) is used most routinely with chronic Q fever defined by presence of anti-phase 1 antibodies [7]. An IgG anti-phase 1 titre of over 800 is one of the Dutch consensus criteria for proven chronic Q fever [7]. PCR can detect *C. burnetti* DNA in serum and can be utilized to make an earlier diagnosis before serology becomes positive [19]. PCR can also be used to detect *C. burnetti* DNA on tissue, including valvular tissue [20]. Culture of *C. burnetti* is used rarely because it lacks sensitivity and poses a biosafety risk and thus is a technique now confined to a few laboratories, such as the Australian Rickettsial Reference Laboratory [21, 22]

The diagnosis of chronic Q fever endocarditis in this case was made more challenging by the presence of a second pathology. A previous case series describes three patients who were also diagnosed with chronic Q fever-related dual-pathogen infective endocarditis [14]. The concomitant bacterial pathogens reported were *Streptococcus salivarius *, *Streptococcus mitis* and *Staphylococcus aureus* in each case, respectively [14]. A similar natural pattern of presentation to that of our patient was described, with an initial alternative bacterial cause for infective endocarditis being found but then the patient having a symptomatic relapse despite appropriate antimicrobial therapy and a subsequent diagnosis of chronic Q fever being made [14]. A previous French retrospective study also describes four patients with infective endocarditis who also had coinfection with *C. burnetti* [23]. One of these cases was also with a viridans group streptococci [23]. Only one of these patients had a history suggestive of chronic Q fever and the diagnosis was delayed in all four cases [23].

There have been other cases of patients being diagnosed with Q fever endocarditis only after their cardiac surgery, as they did not present with typical symptoms; whilst the gross appearance of valves can look normal, histopathology showing acute inflammation has previously prompted clinicians to request Q fever serology to reach the diagnosis [24, 25]. A retrospective study for 20 patients with culture-negative infective endocarditis who went on to have cardiac surgery had Q fever PCR added to tissue samples, and 8 (40 %) of these returned a positive PCR result [20]. Histological examination, when available, showed multinucleated giant cells without a fibrin ring, which was not seen in the Q fever PCR-negative group [20]. Vegetations can be small or absent in Q fever and inflammation may not be significant or nonspecific, therefore performing Q fever PCR on valve tissue should be considered in patients who have epidemiological risk factors for chronic Q fever or who live in a highlighted endemic area, such as Queensland, Australia [26] . Furthermore, if acute inflammation or other abnormal findings are seen on histology and the valve remains culture-negative, clinicians should consider a diagnosis of chronic Q fever and consider further serology or nucleic acid detection.

## Conclusion

Our case highlights the importance of screening patients with infective endocarditis for co-existing Q fever, particularly those considered to have epidemiological risk factors, or a history of unexpected valvular heart disease. Patients undergoing cardiac surgery should have an assessment of their epidemiological risk factors for Q fever and consideration given to testing for Q fever, especially in the presence of acute inflammation or vegetations on histology. Serology and nucleic acid detection can both be used to confirm the diagnosis. Timely diagnosis of Q fever infective endocarditis allows for earlier treatment and may reduce associated morbidity and mortality.
